# Harnessing nanostructured systems for improved treatment and prevention of HIV disease

**DOI:** 10.1002/btm2.10096

**Published:** 2018-07-27

**Authors:** Maya Monroe, Charles Flexner, Honggang Cui

**Affiliations:** ^1^ Dept. of Chemical and Biomolecular Engineering The Johns Hopkins University, 3400 N Charles Street Baltimore MD 21218; ^2^ Div. of Clinical Pharmacology and Infectious Diseases Johns Hopkins University School of Medicine and Bloomberg School of Public Health Baltimore MD 21205; ^3^ Institute for NanoBioTechnology The Johns Hopkins University, 3400 N Charles Street Baltimore MD 21218; ^4^ Dept. of Oncology, Sidney Kimmel Comprehensive Cancer Center The Johns Hopkins University School of Medicine Baltimore MD 21205; ^5^ Center for Nanomedicine The Wilmer Eye Institute, The Johns Hopkins University School of Medicine Baltimore MD 21231

**Keywords:** nanoformulation, HIV treatment, HIV prevention, long‐acting

## Abstract

Combination antiretroviral therapy effectively controls human immunodeficiency virus (HIV) viral replication, delaying the progression to acquired immune deficiency syndrome and improving and extending quality of life of patients. However, the inability of antiretroviral therapeutics to target latent virus and their poor penetration of viral reserve tissues result in the need for continued treatment for the life of the patient. Side effects from long‐term antiretroviral use and the development of drug resistance due to patient noncompliance are also continuing problems. Nanostructured systems of antiretroviral therapeutics have the potential to improve targeted delivery to viral reservoirs, reduce drug toxicity, and increase dosing intervals, thereby improving treatment outcomes and enhancing patient adherence. Despite these advantages, very few nanostructured antiretroviral delivery systems have made it to clinical trials due to challenges in preclinical and clinical development. In this context, we review the current challenges in HIV disease management, and the recent progress in leveraging the unique performance of nanostructured systems in therapeutic delivery for improved treatment and prevention of this incurable human disease.

## 1 INTRODUCTION

Acquired immune deficiency syndrome (AIDS) was first described in 1981.^1^, ^2^, ^3^, ^4^ Human immunodeficiency virus (HIV) was isolated and identified in 1983 before being definitively linked to AIDS in 1984.^1^, ^2^, ^3^, ^4^ HIV targets CD4+ T lymphocytes, leading to a decline in their numbers that ultimately causes the immune dysfunction known as AIDS.^3^, ^5^ As of 2016, 36.7 million people are infected with HIV, with 1.0 million people dying and 1.8 million new infections occurring each year.^6^ This represents a 48% decline in deaths from a peak of 1.9 million in 2006, believed to be a result of increased efficacy of and access to antiretroviral (ARV) therapeutics.^6^

While treatment of HIV infected individuals with three drug combinations of food and drug administration (FDA) approved ARV therapeutics has been extremely effective at controlling viral suppression and preventing the infection from progressing to AIDS, as demonstrated by the global decline in HIV related deaths, it does not clear the virus from the infected individual and must be continued for the lifetime of the patient.^5^, ^6^, ^7^, ^8^, ^9^, ^10^, ^11^ This lack of a cure is attributed to the inability of current therapeutics to target inactive viruses and possibly to limited accumulation in high enough concentrations in viral reservoir tissues.^5^, ^9^, ^10^, ^11^, ^12^, ^13^ Further drawbacks associated with long‐term use of current HIV therapeutics are the need for frequent dosing (once or twice daily), development of resistance due to patient noncompliance, significant adverse effects, and related toxicities.^7^, ^8^, ^9^, ^10^, ^11^, ^14^, ^15^ Consequently, there is an unmet need for a targetable, long‐acting therapeutic delivery system that can increase the time between dosages, reduce fluctuation of drug levels, and increase delivery of the therapeutic agent to viral reservoirs. Nanostructured delivery systems of current ARV drugs have shown promise at addressing these issues but few have progressed to clinical trials due to challenges in preclinical and clinical development.^11^, ^14^

HIV is a lentivirus known for delayed onset and chronic infection with a double‐stranded RNA genome of 9,300 base pairs.^5^, ^16^ The HIV RNA genome contains three major open reading frames: *gag, pol*, and *env* and six small genes that encode regulatory proteins.^5^, ^16^ The *gag* encoded protein is split to form the structural components of the virus.^5^, ^16^ The *pol* encoded polyprotein contains the viral enzymes reverse transcriptase, integrase, and protease, all of which are critical for successful viral infection of host cells.^5^, ^16^ The *env* gene produces the transmembrane protein responsible for viral binding and entry to human immune cells.^5^, ^16^ Each HIV particle consists of a nucleocapsid core that encapsulates two copies of the viral genome and the critical viral enzymes, and that is itself surrounded by a lipid envelope formed from the plasma membrane of the host cell.^5^, ^17^ There are two distinct species of HIV, designated as HIV‐1 and HIV‐2.^5^, ^14^, ^16^ HIV‐1 is the more prevalent virus globally whereas HIV‐2 is more commonly found in West Africa and is believed to progress to AIDS more slowly than HIV‐1.^5^, ^14^, ^16^

**Figure 1:**
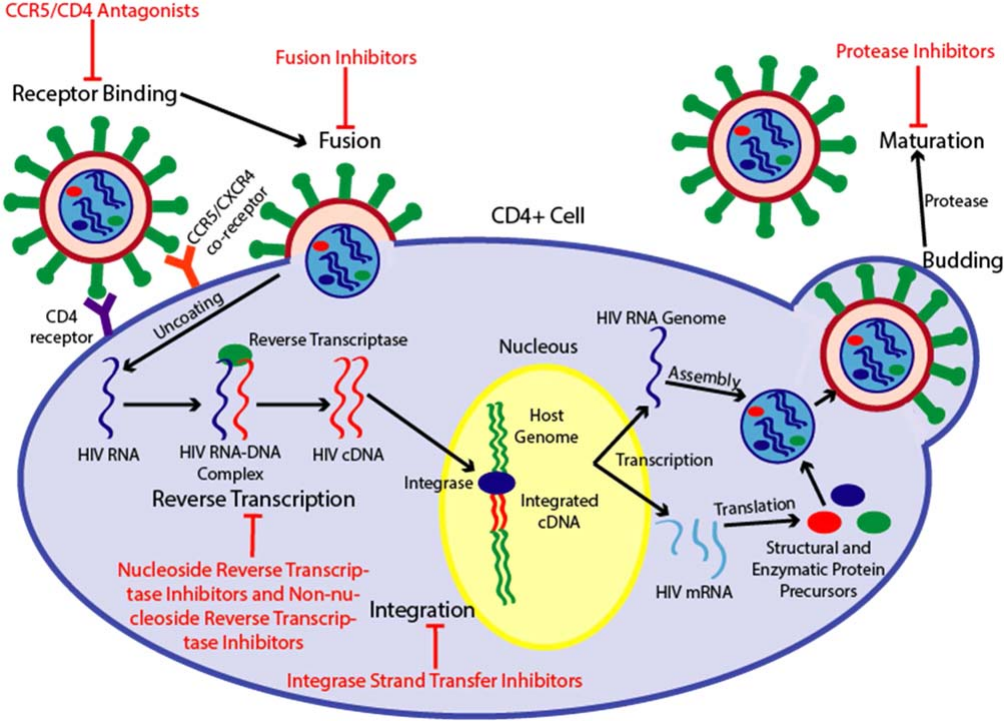
Depiction of the HIV life cycle and the mechanisms of action of the six classes of antiretroviral therapeutics. Mature HIV particles bind with CD4 receptors and either a CCR5 or CXCR4 co‐receptor, triggering a conformational change in the gp41 portion of the HIV *env* protein that enables fusion of the viral and cellular membranes. CCR5 and CD4 antagonists competitively bind with the host cell receptors, preventing viral binding and subsequent entry. Fusion inhibitors bind with gp41, preventing the conformational change needed for cellular entry. Once the viral membrane has fused with the cellular membrane, viral RNA and enzymes are released into the CD4+ cell, where viral reverse transcriptase transcribes the viral RNA into double‐stranded HIV cDNA. Nucleoside reverse transcriptase inhibitors competitively bind with reverse transcriptase and are incorporated into the DNA strand in place of native nucleotides, resulting in chain termination. Non‐nucleoside reverse transcriptase inhibitors non‐competitively bind with a distal hydrophobic pocket in HIV‐1 reverse transcriptase, inducing a conformation change that drastically reduces enzyme activity. The fully synthesized cDNA is incorporated into the host genome by viral integrase, a process that integrase strand transfer inhibitors impede by preventing the formation of covalent bonds between host and viral DNA. Once incorporated into the genome, normal cellular pathways transcribe the cDNA into a full length viral RNA genome and HIV mRNA strands that are translated into precursors of structural and enzymatic viral proteins. The precursor proteins and HIV RNA genome assemble into a viral capsid that buds from the cell, triggering viral protease to cleave the precursor peptides, resulting in full maturation of the viral particle. Protease inhibitors prevent this proteolytic cleavage, which is necessary for the complete development of the viral particle into a mature infectious agent.

The endocytic pathways of HIV invasion, replication and spread are now well understood. The envelope protein gp160 (*env*) of HIV is responsible for HIV's tropism for immune cells.^5^ Env is split into two domains, gp120 and gp41.^18^ Gp120 is a surface protein responsible for binding to the CD4 receptor and either the CCR5 or CXCR4 co‐receptors found on the surface of helper T‐Cells and other lymphocytes and macrophages, which is necessary for cellular entry.^5^, ^16^, ^17^ Gp41 regulates fusion of the viral envelope with the host cell membrane by undergoing a structural change once the viral particle is bound to the host cell, thereby allowing entry.^5^, ^10^ Once the HIV RNA genome is inside the cell, reverse transcriptase utilizes the RNA strands as templates to create an RNA‐DNA duplex of original viral genome and its DNA replica.^5^, ^10^, ^17^ HIV reverse transcriptase is inaccurate and lacks a proof reading function, leading to an average of three incorrect base pairs for each full length viral RNA copied.^5^ The RNA templates are then degraded and the DNA replicas are used to make double‐stranded DNA copies of the viral genome.^5^, ^10^, ^17^ Following transportation into the nucleus, this double‐stranded cDNA replica is inserted into the host genome by viral integrase.^5^, ^10^, ^17^ The integrated viral cDNA is transcribed to create both the complete viral RNA genome and mRNAs that code for the necessary structural, enzymatic, and regulatory proteins of HIV.^5^, ^10^, ^17^ The mRNAs are translated and processed into the viral protein precursors that assemble into immature HIV particles at the cell membrane: the structural protein precursors form around the viral RNA genome, reverse transcriptase, integrase, and protease.^5^, ^10^, ^17^ These protein precursors are cleaved by viral protease as the immature virus is escaping from the cell via budding, completing the formation of a mature viral particle.^5^, ^10^, ^17^, ^19^ The understanding and elucidation of the HIV life cycle has led to the development of six classes of ARV agents with distinct action mechanisms that block HIV activities at different stages (Figure 1).

## 2 CURRENT HIV ARV TREATMENT

Combination antiretroviral therapy (CART), also known as high activity antiretroviral therapy, is currently the standard treatment for HIV‐1 infected individuals.^5^, ^8^, ^9^, ^10^, ^11^, ^14^, ^20^ A minimum of three ARV drugs are used in combination with one another in order to suppress the viral load of a patient to virtually undetectable levels (defined as <50 copies of virus/mL of blood).^5^, ^8^, ^9^, ^10^, ^11^, ^14^, ^20^, ^21^ There are currently 27 FDA approved ARV therapeutics and 1 FDA approved pharmacokinetic enhancer meant to be used to improve the efficacy of ARVs (Tables 1, 2, 3).^22^ ARV therapeutics are divided into six classes: nucleoside/nucleotide reverse transcriptase inhibitors (NRTIs), non‐nucleoside reverse transcriptase inhibitors (NNRTIs), protease inhibitors (PIs), fusion inhibitors (FIs), CCR5 and CD4 antagonists, and integrase strand transfer inhibitors (INSTIs).^5^, ^10^, ^20^, ^22^

**Table 1:** FDA approved nucleoside reverse transcriptase inhibitors for the treatment of HIV

Generic name (abbreviation)	Brand name	Manufacturer	IC‐50 (nM)	Bioavailability (%)	Elimination half‐life (hr)	Plasma protein binding (%)	CSF‐plasma ratio (%)	Dosage form	Adult dosage	Approval year
Abacavir (ABC)	Ziagen	ViiV Healthcare	70–5,800	83	1.54 ± 0.63	∼50	30	Tablet, oral solution	300 mg twice daily or 600 mg once daily	1998
Didanosine (ddl or enteric‐coated didanosine ddl EC)	Videx	Bristol‐Myers Squibb	10–10,000	42	1.5 ± 0.4	<5	21	Powder (oral solution)	125–200 mg twice daily or 250–400 mg once daily	1991
	Videx EC	Bristol‐Myers Squibb	10–10,000	42	1.19 ± 0.21	<5	21	capsule	200–400 mg once daily	2000
Emtricitabine (FTC)	Emtriva	Gilead Sciences, Inc	2–30	93 (capsule) 75 (solution)	∼10	<4	26	Capsule, oral solution	200 mg once daily (capsules) 240 mg once daily (solution)	2003
Lamivudine (3TC)	Epivir	ViiV Healthcare	2–670	86 (tablet) 87 (solution)	5–7	<36	15	Tablet, oral solution	150 mg twice daily or 300 mg once daily	1995
Stavudine (d4T)	Zerit	Bristol‐Myers Squibb	9–4,000	86.4	1.6 ± 0.23	<5	40	Capsule, powder	30–40 mg twice daily	1994
Tenofovir disoproxil fumarate (TDF)	Viread	Gilead Sciences, Inc	2–7	25	∼17	7.2	5	Powder, tablet	300 mg once daily	2001
Tenofovir alafenamide (TAF)	Vemlidy	Gilead Sciences, Inc	5 (EC_50_)	80	0.51 (plasma) 150–180 (cellular)	80	ND	Tablet	25 mg once daily	2016 (for combo therapy)
Zidovudine (AZT or ZDV)	Retrovir	ViiV Healthcare	10–48	64	0.5–3	<38	60	Capsule, sirup, injection	300 mg twice daily (oral) or 1 mg per kg every 4 hr (infusion)	1987

ND = not determined; UD = undetectable.^5^, ^22^, ^23^, ^24^, ^25^, ^26^

**Table 2:** FDA approved protease inhibitors and associated pharmacokinetic enhancers for the treatment of HIV

Generic name (abbreviation)	Brand name	Manufacturer	IC‐50 (nM)	Bioavailability (%)	Elimination half‐life (hr)	Plasma protein binding (%)	CSF‐plasma ratio (%)	Dosage form	Adult dosage	Approval year
Atazanavir (ATV)	Reyataz	Bristol‐Myers Squibb	2–15	ND	∼7	86	<3	Capsule, oral powder	400 mg once daily or 300 mg with 100 mg ritonavir once daily	2003
Darunavir (DRV)	Prezista	Janssen Pharmaceuticals, Inc.	1–5	37 (alone) 82 (with ritonavir)	∼15	95	0.3‐1.8	Oral suspension, tablet	800 mg with 100 mg ritonavir once daily (treatment naïve) or 600 mg with 100 mg ritonavir twice daily (treatment experienced)	2006
Fosamprenavir (FOS‐APV or FPV)	Lexiva	ViiV Healthcare	12–410	ND	7.7	90	12	Oral suspension, tablet	700 mg with 100 mg ritonavir twice daily	2003
Indinavir (IDV)	Crixivan	Merck and Co., Inc.	25–100 (IC_95_)	60–65	1.8 ± 0.4	60	14.7	Capsule	800 mg three times daily	1996
Nelfinavir (NFV)	Viracept	Agouron	7–196 (EC_95_)	20–80	3.5–5	>98	UD	Oral powder, tablet	1250 mg twice daily or 750 mg three times daily	1997
Ritonavir (RTV)	Norvir	AbbVie Inc.	4–150	Absolute: ND >60 based on animal studies	3–5	98–99	<0.5	Capsule, tablet, oral solution, oral powder	600 mg twice daily	1996
Saquinavir (SQV)	Invirase	Hoffman‐La Roche	3.5–10	4	1–2	98	<0.5	Capsule, tablet	1000 mg with 100 mg ritonavir twice daily	1995
Tipranavir (TPV)	Aptivus	Boehringer Ingelheim	30–70	ND	6	>99.9	ND	Capsule, oral solution	500 mg with 200 mg of ritonavir twice daily	2005
Cobicistat (COBI)^a^	Tybost	Gilead Sciences, Inc	ND	ND	3–4	97–98	ND	Tablet	150 mg once daily	2014

ND = not determined; UD = undetectable.

a Pharmacokinetic Enhancer.^5^, ^22^, ^27^, ^28^, ^29^, ^30^, ^31^

**Table 3:** FDA approved non‐nucleoside reverse transcriptase inhibitors, including Efavirenz, Etravirine, Nevirapine, and Rilpivirine

Generic name (abbreviation)	Brand name	Manufacturer	IC‐50 (nM)	Bioavailability (%)	Elimination half‐life (hr)	Plasma protein binding (%)	CSF‐plasma ratio (%)	Dosage form	Adult dosage	Approval year
Efavirenz (EFV)	Sustiva	Bristol‐Myers Squibb	3–9	50	52–76	99.5	0.26–1.19	Capsule, tablet	600 mg once daily	1998
Etravirine (ETR)	Intelence	Janssen Pharmaceuticals, Inc.	1–5	ND	∼41	99.9	ND	Tablet	200 mg twice daily	2008
Nevirapine (NVP and extended‐release nevirapine NVP XR)	Viramune	Boehringer Ingelheim	10–100	93 (tablet) 91 (solution)	25 ‐ 30	∼60	45	Tablet, oral suspension	200 mg twice daily	1996
	Viramune XR	Boehringer Ingelheim	10–100	80–94	25–30	∼60	45	Tablet	400 mg once daily	2011
Rilpivirine (RPV)	Edurant	Janssen Pharmaceuticals, Inc.	42	ND	∼50	99.7	ND	Tablet	25 mg once daily	2011
Enfuvirtide (T‐20)	Fuzeon	Hoffman‐La Roche; Genentech	0.1–1,700	84 (subcutaneous injection)	3.8 ± 0.6	92	UD	Subcutaneous injection	90 mg twice daily	2003
Ibalizumab	Trogarzo	TaiMed Biologics USA Corp.	53	NA	2.7–64 (dose dependent)	ND	ND	Intravenous injection	800 mg every two weeks	2018
Maraviroc (MVC)	Selzentry	ViiV Healthcare	0.1–4.5	23–33	14–18	76	2.8	Tablet, oral solution	150, 300, or 600 mg twice daily (depending on combination therapeutics)	2007
Bictegravir (BIC)	Biktarvy	Gilead Sciences, Inc	7.5	ND	14.9–20.9	>99	ND	Tablet	50 mg once daily (in combination with 200 mg FTC and 25 mg TAF)	2018 (for combo therapy)
Elvitegravir (EVG)	Vitekta	Gilead Sciences, Inc	44	ND	8.7	98–99	ND	Tablet	85 or 150 mg once daily (depending on combination therapeutics)	2014
Dolutegravir (DTG)	Tivicay	ViiV Healthcare	2.7–12.6	ND	14	98.9	0.11–0.66	Tablet	50 mg once daily or 50 mg twice daily (depending on past and combinedtherapeutics)	2013
Raltegravir (RAL)	Isentress	Merck and Co., Inc.	6–50 (IC_95_)	ND	9	83	5.8	Tablet, oral suspension	400 mg twice daily or 1200 mg once daily	2007
	Isentress HD	Merck and Co., Inc.	6–50 (IC_95_)	ND	9	83	5.8	Tablet	400 mg twice daily or 1200 mg once daily	2017

Fusion inhibitors (Enfuvirtide); CCR5 and CD4 Antagonists (Ibalizumab and Maraviroc); and Integrase strand transfer inhibitors (Bictegravir, Elvitegravir, Dolutegravir, and Raltegravir) for the treatment of HIV.

NA = not applicable; ND = not determined; UD = undetectable. 5, 22, 32, 33, 34, 35

### 2.1 ARVs mechanisms of action

**Figure 2:**
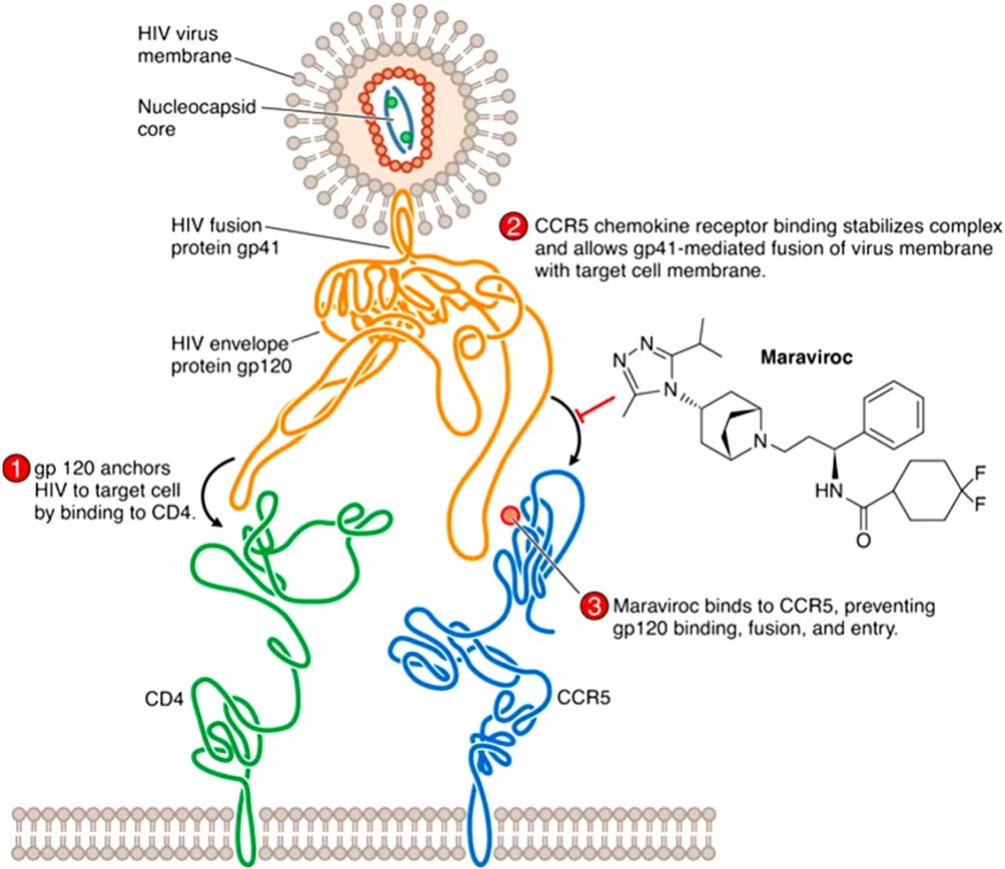
Mechanism of action for the CCR5 antagonist maraviroc. The drug competitively binds to the host cell's CCR5 receptors, preventing the gp120 portion of the HIV *env* protein from binding to the co‐receptor. By disrupting the interaction between gp120 and CCR5 necessary for fusion of the viral and cellular membranes, CCR5 antagonists prevent cellular entry. Used with permission from Ref. 5.

NRTIs competitively bind with reverse transcriptase when the viral DNA strand is being synthesized, resulting in the addition of an NRTI rather than a native nucleotide.^5^, ^10^ This results in chain termination of the cDNA because NRTIs lack the 3′ OH group necessary for the covalent attachment of another nucleotide to the strand.^5^, ^10^ NNRTIs non‐competitively bind to a distal hydrophobic pocket in HIV‐1 reverse transcriptase, inducing a conformational change that disrupts the catalytic site of the enzyme and drastically reduces reverse transcriptase activity.^5^, ^10^ PIs prevent the cleavage of gag and pol precursor polypeptides at the phenylamine‐proline target peptide sequence.^5^, ^10^, ^36^ If the precursor polypeptides are not cleaved then the virus particles are incapable of developing into mature viral particles.^5^, ^10^, ^36^ FIs are a type of entry inhibitor that disrupt the fusion of the viral and cell membranes by binding to the gp41 domain of env, preventing a necessary structural change that enables fusion.^5^, ^10^ CCR5 and CD4 antagonists are unusual in their mechanism of action because they interfere with host cell receptors rather than viral components.^5^ They competitively bind to the host cell CCR5 co‐receptor or CD4 receptor, preventing binding of HIV gp120 and subsequent fusion and entry (Figure 2).^5^, ^10^ CCR5 tropic HIV utilizes CCR5 as its binding co‐receptor with CD4 rather than CXCR4.^5^ CCR5 tropic HIV is responsible for new infections, with all HIV isolated in the early stages of infection identified as CCR5 tropic.^37^ INSTIs block the integration of viral DNA into host DNA by binding to HIV integrase and preventing the formation of covalent bonds between host and viral DNA, thereby blocking strand transfer into the host genome.^5^, ^10^

### 2.2 FDA therapeutic standards

As per the FDA guidelines on HIV treatment, CART consists of a combination of at least three ARVs from at least two different classes.^8^, ^10^, ^20^ The recommended HIV CART regimen utilizes two NRTIs in combination with an INSTI, NNSTI, or pharmacokinetically enhanced PI (Table 4).^20^ The goal of ARV therapy is to suppress viral replication to the greatest extent possible and for the longest time possible.^5^, ^20^ When used as recommended, CART is extremely effective at reducing viral load; it suppresses viral replication in more than 80% of those beginning treatment.^5^, ^8^, ^9^, ^10^, ^11^, ^14^, ^20^ Plasma viral load (the number of HIV RNA copies detected per ml of a patient's blood) is the best stand‐alone, prognostic marker for progression to AIDS.^38^ Furthermore, it has been definitively linked to the efficiency of viral transmission from infected individuals to healthy individuals, with infected individuals with viral loads below 500 copies/mL being extremely unlikely to transmit the virus to others.^10^, ^39^

**Table 4:** FDA recommended initial regimens of CART^20^

Preferred	INSTI + 2 NRTIs
regimens	Dolutegravir, Abacavir, Lamivudine
	Dolutegravir, Tenofovir, Emtricitabine
	Elvitegravir, Cobicistat, Tenofovir, Emtricitabine
	Raltegravir, Tenofovir, Emtricitabine
Recommended in certain situations	Boosted PI (either Cobicistat or Ritonavir) + 2 NRTIs
	Darunavir or Atazanavir, Tenofovir, Emtricitabine
	Darunavir or Atazanavir, Abacavir, Lamivudine
	NNRTI + 2 NRTIs
	Efavirenz or Rilpivirine, Tenofovir, Emtricitabine
	INSTI + 2 NRTIs
	Raltegravir, Abacavir, Lamivudine
	Regimens if Abacavir and Tenofovir cannot be used
	Darunavir, Raltegravir
	Lopinavir, Lamivudine

### 2.3 Shortcomings of CART

**Figure 3:**
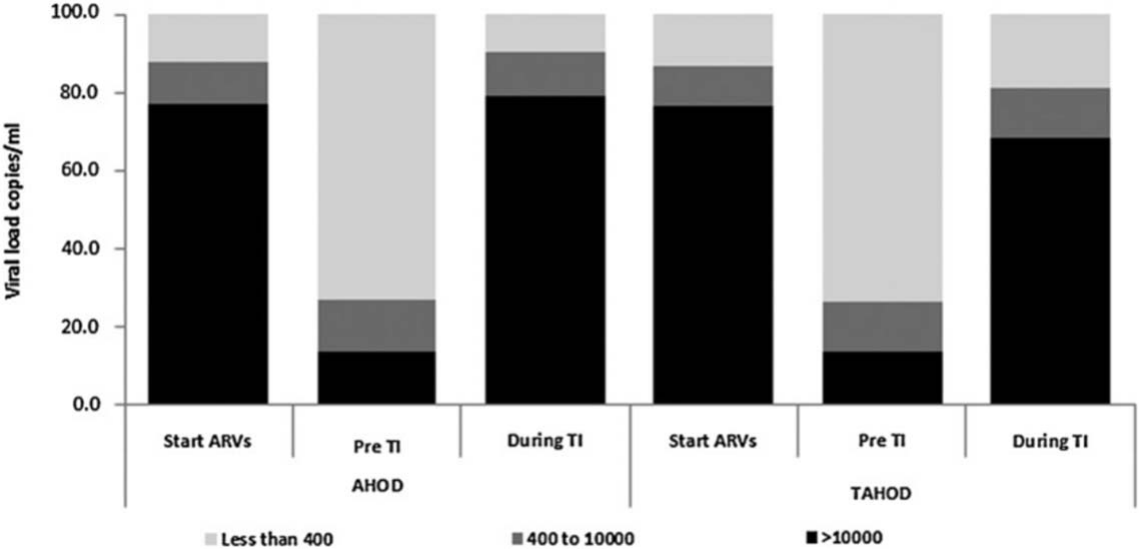
The percent of individuals with viral loads <400 copies/mL, 400–10,000 copies/mL, and >10,000 copies/mL in the Australian HIV Observational Database (AHOD) and Treat Asia HIV Observation Database (TAHOD) at the start of therapy, prior to a treatment interruption (TI) consisting of the discontinuation of ARV therapeutics for >30 days, and during the TI. Used with permission from Ref. 44.

While CART has demonstrated success at suppressing the viral load of new patients, thereby delaying the progression to AIDS and decreasing the chance of viral transmission, CART is unable to clear HIV from infected individuals.^5^, ^8^, ^9^, ^10^, ^11^, ^14^ Consequently, there is no cure for HIV and CART must be continued for the life of the patient.^5^, ^8^, ^9^, ^10^, ^11^, ^14^ Discontinuation of the ARV therapy leads to a rapid spike in viral load and the development of drug resistance (Figure 3).^5^, ^8^, ^9^, ^10^, ^11^, ^14^, ^15^ The continued presence of HIV in infected individuals despite CART causes this viral rebound and is believed to be a result of latent viral DNA found in quiescent cells, and possibly active HIV infected cells in physiological compartments that ARVs do not easily penetrate.^5^, ^9^, ^10^, ^11^, ^12^, ^13^ Currently approved ARVs are incapable of targeting the latent viruses because their mechanisms of action are dependent on viral replication.^5^ However, the presence of residual viral reserves in tissues that CART does not efficiently enter (such as the lymphoid tissues, the central nervous system (CNS) and the testis) could be addressed by targeted delivery of approved ARVs to these reserves. The concentrations of ARV drugs in lymph node mononuclear cells (LNMCs) have been shown to be 66–99% lower than their respective concentrations in peripheral blood mononuclear cells (PBMCs).^12^ Residual viruses isolated from these reserve tissues are still known to be responsive to the CART regimen of the patient.^9^, ^40^, ^41^ This indicates that the continued existence of these active viruses may be due to insufficient concentrations of ARV therapeutics in the tissue rather than acquired resistance to the drugs, especially since sequencing of the recovered viral genome revealed no known drug resistance sequences.^42^ Therefore, in order to better control HIV infections and potentially eliminate HIV from the body, treatments that target these viral reservoirs could be explored.

Furthermore, ARVs are known to have significant adverse side effects, toxicity due to off‐target binding or accumulation, and common and often complicated interactions with other drugs.^5^, ^8^, ^9^, ^10^, ^11^, ^14^, ^20^, ^22^ CART requires once or twice daily doses for the lifetime of the patient, thereby increasing the likelihood that patients will develop serious adverse effects due to long‐term use and making patient compliance to the regimen difficult.^9^, ^11^, ^15^ Up to 20% of the HIV infected population in the United States has failed to attain full HIV viral load suppression (<200 copies/mL) under sustained CART,^43^ a problem that is attributed to patient noncompliance to the treatment regimen.^6^, ^15^ Patients with difficulty swallowing such as children, others with neurologic, gastrointestinal, or psychiatric disease, and intravenous drug users are at particular risk of not adhering to this self‐administered, oral regimen.^9^, ^15^ Fluctuating levels of ARVs in the body can lead to a rebound in viral load and the development of drug resistance.^11^, ^14^, ^15^ It is the rapid mutation of HIV due to the inaccuracy of reverse transcriptase that causes this sudden drug resistance and that makes the development of a broadly functional HIV vaccine difficult.^5^, ^8^ Viral replication must be suppressed to prevent the formation of mutated viruses that may contain novel resistance mutations to the administered therapy.^11^ While inconsistent ARV concentrations are often due to patient failure to take medications as prescribed,^11^, ^15^ it is also possible that currently available formulations of ARVs cannot sustain drug concentrations at the necessary levels for the desired dosing interval.^14^

A better, long‐acting, targetable delivery system is needed to increase the intervals between doses, limit fluctuation of medication levels in between doses, reduce the side effects and toxicity associated with off‐site delivery, and increase the concentration of ARV drugs delivered to HIV viral reserve tissues. HIV infected patients have shown interest in less frequent dosage administration: 73% of HIV infected patients surveyed had a favorable response to less frequent intravenous doses, a number that rises to 84% if the intravenous doses can be administered monthly.^45^ In order to address this desire for longer dosing intervals, there has been an increase in the investigation and development of drugs with longer terminal half‐lives that could be orally administered once a week.^5^, ^46^ However, such new ARVs tend to be expensive and increased access to effective therapies can better be obtained through a delivery system designed to extend the circulation and reduce the toxicity of older, generically available drugs.^14^ Furthermore, such long‐acting oral formulations do not address the issues of targeting to the viral reserve tissues and only a few drugs exhibit long enough half‐lives, effectively limiting the combination therapies possible.^15^ Nanostructured delivery systems for traditional ARVs are a potential strategy to address the issues of toxicity, resistance, and insufficient accumulation in HIV viral reserve tissues affiliated with CART.

## 3 NANOSTRUCTURED SYSTEMS FOR THE TREATMENT OF HIV

**Figure 4:**
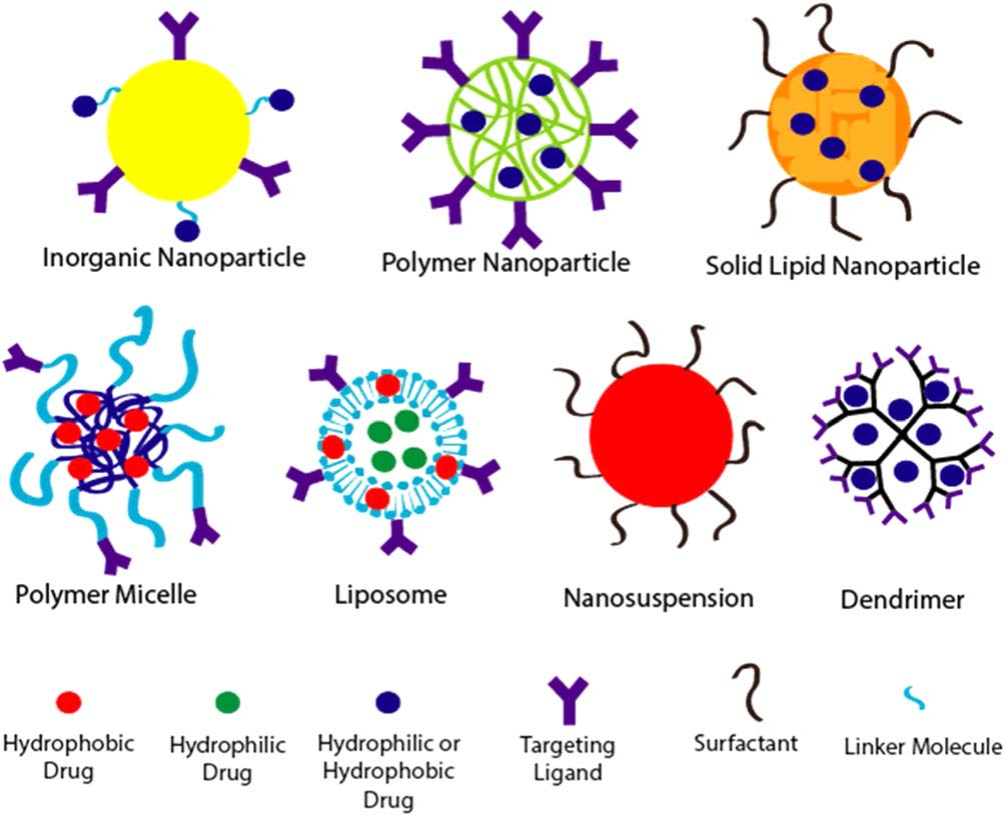
Commonly used nanostructured systems for the investigational delivery of HIV antiretroviral therapeutics. Inorganic NPs have gained popularity due to the inherent antiviral activity of some materials such as silver. Liposomes and solid lipid NPs have also experienced increased use as a result of their ability to permeate the BBB.

Nanostructured ARV drug delivery systems have been explored for both systemic treatment to control HIV replication in infected individuals and for local delivery to prevent the acquisition of the infection. When therapeutics are delivered via nanoparticles (NPs), the nanosystem for the most part governs the pharmacokinetic profile rather than the drug itself.^47^, ^48^ Nanoscale delivery systems of various types are advantageous in that they can be created with a variety of physicochemical properties to control drug release, protect the drug from metabolic break down and clearance from the body, enable targeting of specific types of cells or tissues, limit adverse side effects and toxicity, and maintain constant therapeutic concentrations.^10^, ^47^ Furthermore, NPs are known for their ability to deliver therapeutics to regions of the body that are not otherwise attainable.^49^ Nanotherapeutic platforms typically used in HIV ARV delivery include polymer, inorganic, and solid lipid NPs, polymer micelles, liposomes, nanosuspensions, and dendrimers (Figure 4). Initially, nanostructured delivery systems were designed to increase the plasma half‐lives of ARVs in order to create a long‐acting therapy.^7^, ^10^, ^11^ While longer dosing intervals would be well received by patients and could lead to improved adherence,^15^, ^45^ these systems do not necessarily result in improved targeting of viral reservoirs.^9^

### 3.1 Targeted systemic delivery systems

The inability of CART to fully eradicate the virus from the body is in part attributed to the limited concentration of ARVs present in the lymphatic tissues and other viral reserve tissues.^7^, ^12^ In order to fully eliminate these viral reservoirs, the ARV concentrations in the lymphatic and central nervous systems must be maintained at effective levels. This requires not just the use of nanoscale systems for ARV delivery, but also the ability of these systems to target the drugs to HIV viral reservoirs, especially the CNS and lymph nodes. These tissues pose unique challenges for drug accumulation due to the exclusivity of the blood brain barrier (BBB), which tightly controls the transport of substances into the CNS,^50^ and the rapid rate at which small molecules diffuse out of the lymph and back into the blood.^9^, ^51^

#### 3.1.1 Inorganic NPs

Inorganic materials such as copper, iron, silver, gold, or silica are used to form inorganic NPs and the therapeutic agents are conjugated to the surface along with any desired peptides or antibodies for functionalization. Gold nanoparticles (AuNPs) have been commonly used in HIV nanotherapy because of their biocompatibility.^52^ Silver nanoparticles (AgNPs) are of particular interest in the treatment of HIV due to their innate antiviral activity, thereby enabling the NP to act as an HIV therapeutic itself.^53^ AgNPs between 1 and 10 nm are believed to inhibit HIV infection by binding to the gp120 protein responsible for viral binding to the CD4 receptor and co‐receptors, preventing cellular entry.^7^, ^54^ However, AgNPs perforate brain micro‐vascular endothelial cells (BMECs) and induce BBB inflammation,^50^ making them unsuitable for use in delivery systems that target the HIV reservoir in the brain.

Some nanostructured systems are focused on iron NPs because their use in magnetic resonance imaging allows for rapid assessment of biodistribution. Surface modification of superparamagnetic iron oxide (SPIO) NPs has been used by Gendelman and coworkers to target the delivery systems to monocytes and macrophages.^55^, ^56^ Immunoglobulin (IgG) was attached to the surfaces of SPIO NPs.^55^ The Fc receptor mediated phagocytosis of IgG coated particles led to a 10‐fold increase in uptake of IgG‐SPIO NPs by monocytes and macrophages in comparison to that of uncoated SPIO NPs.^55^ MRI imaging of the NPs revealed increased accumulation of the IgG‐SPIO NPs in lymphatic tissues such as the spleen.^55^ Folic acid (FA) surface‐modified SPIO NPs achieved similar results by conjugating FA modified polyethylene glycol (PEG) to the SPIO NPs, with a more than twofold increase in accumulation in the spleen (13 µg/mL for FA‐PEG‐SPIO NPs vs. 6 µg/mL for PEG‐SPIO NPs).^56^ ARVs can be conjugated to the surfaces of these targeted SPIO NPs to increase the drug accumulation in the viral reservoirs.^55^, ^56^ In an attempt to target the brain specifically, the Corsi lab developed amphiphilic polymer coated iron oxide NPs to improve the BBB penetration of enfuvirtide (ENF).^57^ Such NPs were found to have 170% better permeability through an *in vitro* double layer of rat BMVECs supported by astrocytes than free ENF, and comparatively higher accumulation in the brains of mice during an *in vivo* study.^57^ The mechanism of penetration by the NPs is believed to be some form of non‐endocytotic transcytosis through the BMECs due to their tendency to attach to the plasma membranes of the cells.^57^

Conjugation to inorganic NPs has been shown by Margolis and coworkers to improve the antiviral activity of ineffective therapeutics. A delivery system with potent anti‐HIV activity can be created from a low‐affinity small molecule that displays no therapeutic activity on its own by conjugating several of the inactive molecules to an AuNP.^58^ Conjugation of SDC‐1721, a biologically inactive derivative of the ARV maraviroc, to 2.0 nm AuNPs at a ratio of 12:1 SDC‐1721 to NPs resulted in the creation of a multivalent AuNP with ARV activity similar to that of maraviroc.^58^ Neither SDC‐1721 or AuNPs exhibited independent suppression of viral replication, but the multivalent SDC‐1721 AuNPs inhibited viral replication with an average IC_50_ value of 10 nM, matching the average IC_50_ value of maraviroc.^58^ The uptake of 2–10 nm AuNPs by macrophages, PBMCs, and human‐brain micro‐vascular endothelial cells (HBMECs) and the accumulation of unmodified AuNPs in the spleen and, to a much lesser extent, the brain, was reported.^59^ By conjugating thiolated raltegravir (RAL), an inactive form of RAL, to AuNPs in a 4:1 ratio, the researchers were able to suppress viral replication to 25.23%.^59^ While free RAL demonstrates better HIV suppression, the RAL‐AuNPs did exhibit better cellular uptake, BBB penetration, and accumulation in the spleen and brain.^59^ The accumulation in viral reservoirs was accompanied by increased off‐site accumulation in the liver, kidneys, lungs, and heart.^59^

Another proposed delivery system of glyceryl monostearate‐modified, nevirapine loaded gold shell NPs displayed similar increased uptake by PBMCs and sustained accumulation in viral reservoirs such as the spleen, lymph nodes, thymus, bone marrow, and brain.^60^ But once again, the improved viral reserve tissue accumulation was accompanied by increased off‐site accumulation in the liver and ovaries.^60^ Such off‐site accumulation of nanomaterials is concerning given the potential cytotoxicity of inorganic NPs, making the use of a more biocompatible system necessary. Additionally, since most inorganic NPs are not biodegradable, their size is often limited to several nanometers in order to facilitate renal clearance.

#### 3.1.2 Liposomes

The superior biocompatibility and ease of functionalization of liposomes make them an attractive alternative to inorganic NPs.^14^ Cationic liposomes are rapidly gaining popularity as delivery vesicles for ARVs due to their ability to penetrate the BBB via adsorption‐mediated transcytosis through BMECs.^50^ They consist of a phospholipid bilayer that encloses an aqueous core and they can be used to transport either hydrophobic drugs dispersed in the phospholipid tails or hydrophilic drugs encapsulated in the aqueous core. Targeting moieties or short strands of PEG can be attached to the surface to direct accumulation in specific tissues or protect the liposomes from immune clearance.

By attaching sugars to the surface of liposomes, it is possible to increase cellular uptake of the encapsulated ARV via carbohydrate binding receptors found on the surface of lymphocytes, monocytes, and macrophages.^61^ In a study conducted by the Jain lab, galactosylated liposomes loaded with zidovudine (ZDV) exhibited increased ZDV uptake by alveolar macrophages *in vitro*.^61^ The sugar modified liposomes also demonstrated increased plasma concentrations, decreased kidney uptake, and higher distribution to the liver, spleen, lymph nodes and lungs by almost 10‐fold higher than free ZDV following intravenous administration in a rat model.^61^ While this study sought to demonstrate the ability of modified liposomes to accumulate in viral reserve tissue, another property that make liposomes an interesting delivery vehicle for ARVs is their transdermal capabilities.^62^ The transdermal delivery of liposomes can be further enhanced by incorporating ethanol into indinavir (IDV) liposomes.^62^ The incorporation of 45% ethanol in liposomes increased the transdermal flux through cadaver skin from 3.2 µg/cm^2^/hr for free IDV drug solution and 6.3 µg/cm^2^/hr for 0% ethanol liposomes to 27.2 µg/cm^2^/hr.^62^ This transdermal delivery system of ARVs would likely be an attractive alternative to daily oral therapy or other nanomedicine platforms administered via injection. Its potential to produce improved macrophage uptake and enhanced drug accumulation in viral reserve tissues, as demonstrated by the earlier mentioned liposomes produced by the same lab, make such a delivery system worthy of further investigation.

An alternative approach to improve BBB penetration of liposomes utilizes the application of an external magnetic field to magnetic liposomes.^63^, ^64^ Arginylglycylaspartic acid (RGD) modified, magnetic liposomes exhibited a 9.1‐fold increase in brain concentration of the encapsulated drug in comparison to free drug, leading to the design of magnetic liposomes that could both directly penetrate the BBB and induce monocyte‐mediated trans endothelial migration across the BBB.^63^, ^64^ The RGD sequence enhanced the uptake of the liposomes by monocytes and neutrophils and the magnetic nature of the liposomes induced the liposome loaded cells to cross the BBB via monocyte/neutrophil‐mediated penetration following the application of an 8.0 kg magnetic field near the brains of rats.^63^ By encapsulating zidovudine triphosphate (ZDVTP), the active form of ZDV produced *in vivo*, and magnetite particles within 150 nm liposomes and applying an external magnetic field, BBB transmigration across a single layer of HBMECs supported by human astrocytes (HAs) was enhanced by almost 300% in comparison to free ZDVTP.^64^ These magnetic liposomes were also capable of uptake by PBMCs, and PBMCs that contained the magnetic liposomes exhibited more than twofold increased penetration of the *in vitro* BBB model upon application of a magnetic field compared to PBMCs that did not contain magnetic liposomes.^64^ The magnetic liposomes did not display cytotoxicity of PBMCs, and the encapsulation of the ZDVTP did not negatively affect the anti‐HIV‐1 efficacy of the drug.^64^ Building on this success, magneto‐plasmonic NPs that consist of gold coated, iron chloride particles encapsulated in tenofovir disoproxil fumarate (TDF) loaded liposomes were developed.^65^ The combination of iron and gold imparts on the hybrid NPs the unique functionalities of both magnetic NPs and AuNPs.^65^, ^66^ These hybrid magneto‐plasmonic liposomes can be easily imaged by different techniques, including MRI and X‐ray computed tomography, and display improved BBB penetration via magnetic targeting with an external magnetic field.^65^ Magneto‐plasmonic liposomes displayed a 1.8‐fold increase in transmigration across an *in vitro* BBB model of HBMECs supported by HAs and pericytes than liposomes that did not contain the magneto‐plasmonic particles.^65^ They also demonstrated an improved therapeutic effect with increased suppression of viral replication in HIV infected microglial cells in comparison to free TDF.^65^

Although magnetic targeting of magnetic liposomes to the viral reserve tissues is possible, it is not necessary to improve accumulation of ARVs at the sites of action, and it complicates the production of the liposomes and the execution of the therapy.^9^, ^67^ The Ho lab was able to demonstrate improved lymph node accumulation of PEGylated liposomes encapsulating three ARV drugs.^9^, ^67^ Hydrophilic tenofovir (TFV) was trapped inside the aqueous core and attached to the hydrophilic heads of the phospholipids, and hydrophobic ritonavir (RTV) and lopinavir (LPV) were stored within the hydrophobic core of the phospholipid bilayer.^9^, ^67^ 24 hr after subcutaneous delivery of the three free ARVs in a primate animal model, virtually no detectable concentration of RTV or LPV was found in LNMCs and 224.2–287.2 ng/mL of TFV was found in LNMCs.^67^ Subcutaneous injection of liposomes encapsulating the ARVs resulted in accumulation of more than 1,200 ng/mL of LPV and 1,600 ng/mL of RTV in the LNMCs.^67^ Liposomal encapsulation did not improve the LNMC concentration of TFV, but it did improve the plasma concentration of the ARV.^67^ While increased lymph node accumulation was demonstrated by attaching CD4 targeting peptides to the surface of the liposomes, such additions add complexity to system and make scale up difficult.^9^ Given the accumulation of unmodified liposomes in the lymph nodes, it is desirable to first pursue the development of the simpler delivery system.^9^ While many nanomedicine platforms have been explored for the delivery of one ARV, very few have encapsulated three ARVs. Since monotherapy is not an option for treatment, and all recommended initial HIV regimens consist of a minimum of three anti‐HIV therapeutics, it is extremely important for clinical development that combination nanotherapeutics be investigated. While the combination of RTV, LPV, and TFV is not currently recommended as a therapy by the FDA,^20^ the delivery system proposed by the lab could be adjusted to incorporate other ARV combinations. However, the poor storage stability and limited loading capacity of liposomes must be addressed if they are to have true clinical potential.^14^

#### 3.1.3 Solid lipid NPs

Solid lipid nanoparticles (SLNs) are a type of colloidal NP composed of physiologically solid lipids dispersed in an aqueous surfactant solution often containing phospholipids (Figure 4).^68^, ^69^ SLNs exhibit similar biocompatibility to liposomes with better entrapment efficiency of hydrophobic drugs, lower cost, and improved scalablity.^14^, ^69^ They can be used to encapsulate hydrophilic or hydrophobic drugs and the surface can be modified with ligands for tissue targeting.^68^, ^70^ The properties and therapeutic release of the SLNs can be controlled by changing the lipid components and surface modifications.^68^ Encapsulation efficiency of hydrophilic substances in SLNs is generally low unless double emulsion formulation is used, but trace solvent residues from this technique can cause toxicity.^70^, ^71^ SLNs made with cationic lipids show improved cellular uptake believed to be a result of charge‐facilitated interactions with cellular receptors.^72^ This increased uptake by cells can contribute to increased penetration of the BBB and accumulation of SLNs in the brain.^68^, ^73^

One of the early applications of SLNs to HIV treatment was carried out in 2006. The BBB permeability of three different ARVs, stavudine, delavirdine, and saquinavir (SQV), were compared between two polymeric NPs made of polybutylcyanoacrylate (PBCA) and methylmethacrylate‐sulfoproprylmethacrylate and microemulsion‐prepared, slightly anionic SLNs^74^ All three delivery systems showed improved BBB penetration for all three ARVs, with 3–16‐fold increases in uptake by a monolayer of HBMECs in comparison to uptake of the free drugs.^74^ The positively charged PBCA NPs displayed the best BBB permeability of the three delivery systems for all ARVs encapsulated due to their potential for electrostatic interactions with the negatively charged HBMECs.^74^ In order to improve the permeability of SLNs, novel cationic SLNs were developed.^75^ By incorporating cationic stearylamine and dioctadecyldimethyl ammonium bromide in the lipid components, SQV encapsulating cationic SLNs were created via the microemulsion technique.^75^ The nonionic lipids passively accumulate in the center of the SNL and the cationic lipids are found on the peripheral edges of the lipid core, the ratios of the lipid components can be adjusted to attain the lipid composition with the best SQV entrapment and release profiles.^75^, ^76^ These cationic SLNs were further modified by altering the composition of the nonionic surfactant solution to better bring out their cationic nature.^77^ The surfactant that resulted in the more positively charged SLN surface produced a stronger attraction between the cationic SLNs and HBMECs based on the electrical interaction energy between the NPs and the cells.^77^ HBMEC permeability was improved from 4.3 × 10^−6^ cm/s to > 5.6 × 10^−6^ cm/s by altering the lipid composition of the SLNs to have a higher weight percentage of cholesterol and cationic esterquat 1.^78^ The use of cholesterol as one of the nonionic lipids in the cationic SLN is believed to improve BBB penetration via cholesterol receptor‐mediated transcytosis across the HBMECs.^78^ All of this suggests the highly tunable nature of SLN biodistribution via surfactant and composition alteration.

Tissue distribution studies revealed the potential of SLNs for lymphatic tissue targeting in addition to CNS penetration.^79^ Even nonionic SLNs displayed improved uptake by reticuloendothelial system tissues in comparison to polylactic‐co‐glycolic acid (PLGA)‐poloxamer 188 NPs, with an increased concentration of the loaded ARVs found in the spleen and liver when delivered via SLNs.^79^ SLNs are naturally capable of accumulating in intestinal lymphatic tissues because they induce the formation of chylomicrons by enterocytes, which promotes the absorption of their lipid matrix by the intestinal lymphatic system.^80^, ^81^ The concentration of LPV when administered directly into the duodenum via glycerol behenate SLNs was 4.91 times greater in the intestinal lymph than LPV administered in a methyl cellulose suspension.^80^ Similarly, efavirenz (EFV) encapsulated in multi‐lipid SLNs was found in reduced concentrations in the liver (44.70% lower) and increased concentrations in the spleen in comparison to free EFV.^81^ This indicates that EFV SLNs bypassed the liver by entering the lymph following oral administration in rats.^81^ Even ignoring the potential for BBB penetration and lymphatic targeting, encapsulation of ARVs within SLNs can drastically improve the pharmacokinetics of the drugs.^82^ Loading of EFV in glycerol monostearate SLNs increased the peak plasma concentration of the drug by more than five times that of free EFV and the AUC (area under the curve of the plasma drug concentration versus time) by more than 10‐fold.^82^ Despite these advantages, the limited batch to batch reproducibility, sterilization difficulties, and poor stability of SLNs pose challenges for their widespread use.^14^

#### 3.1.4 Polymeric NPs

Improved stability and reproducibility can be obtained by using polymeric NPs as the delivery system. Polymeric NPs can consist of a polymer membrane that encapsulates a drug reservoir, a NP of polymer matrix with drug evenly distributed throughout, or a solid polymer NP with drugs and targeting moieties conjugated to the surface. The NPs tend to be made of biodegradable polymers such as polylactic acid (PLA), polyglycolic acid (PGA), or a copolymer of the two (PLGA) or to be made of biocompatible polymers such as PEG. They are capable of encapsulating both hydrophilic and hydrophobic drugs and the release profile of the incorporated therapeutics can be tuned by altering polymer structure, particle size, pore size, degradation rate, and conjugation chemistry. The shape and size of the NPs are dependent on production technique rather than polymer structure.

The Kuo lab has demonstrated the improved BBB penetration of ARVs encapsulated within the matrix of surface‐modified PLGA NPs.^83^, ^84^, ^85^ Initially, SQV was entrapped in the PLGA matrix to form SQV‐PLGA NPs.^83^, ^84^ BBB penetration was enhanced by conjugating poly‐γ‐glutamic acid (γ‐PGA), a biosynthesized hydrophilic polymer that has been shown to improve cellular uptake, to the surface of the SQV‐PLGA NPs.^83^ 85.2% grafting efficiency of 6 kDa γ‐PGA increased penetration of the NPs through a monolayer of HBMECs supported by HAs by six times that of free SQV.^83^ Conjugation of polyethyleneimine (PEI) to the ends of the γ‐PGA strands already conjugated to the SQV‐PLGA NPs was shown to decrease the release rate of SQV from the NPs, thereby improving control of the drug release profile, limiting the initial burst release, and prolonging the therapeutic lifetime of the NPs.^84^ The addition of PEI also has the potential to improve BBB penetration due to its cationic nature.^84^ BMECs have negatively charged surfaces, and electrostatic interactions draw positively charged materials to these surfaces, resulting in increased endocytic transport of the materials through the BMECs into the CNS.^84^ Administering these PEI/γ‐PGA/SQV‐PLGA NPs and then applying an electromagnetic field increased endocytosis of the NPs into an HA supported monolayer of HBMECs by 2.38 times the uptake of the NPs administered without the electromagnetic field.^86^ Improved BMEC uptake in comparison to free drug resulted from decorating the surface of nevirapine PLGA NPs with transferrin, a transport vector that has been shown to increase BBB penetration.^85^

Other systems have attempted to penetrate viral reservoirs by targeting macrophages.^87^, ^88^ Macrophages can enter the lymphoid tissue and CNS, thus cellular uptake of ARV NPs could result in improved drug biodistribution by increasing accumulation of ARVs in the reservoir tissues.^87^ Coating materials with FA has demonstrably improved macrophage cellular uptake via highly expressed folate receptors.^87^ Encapsulating dolutegravir and europium‐doped cobalt ferrite in a lipid coated, FA surface‐modified, polycaprolactone matrix led to improved macrophage uptake in comparison to uncoated NPs and sustained ARV activity.^88^ The cobalt ferrite distributed throughout the polymer matrix also enabled the rapid determination of drug biodistribution using MRI. 88 Despite these successes, polymer NPs are hampered by their potential polymer toxicity and their limited scalability, making the investigation of other systems necessary.^14^

#### 3.1.5 Polymer micelles

Above their critical micellar concentration, amphiphilic block copolymers can self‐assemble into micellar aggregates. It is this capability to self‐assemble that differentiates polymer micelles from polymer NPs. Unlike polymer NPs, the molecular design plays an important role in defining the architecture and size of the micelles. Because the self‐assembly is a result of energy minimization, the micelles demonstrate high thermodynamic stability .^14^ Hydrophobic therapeutics can either be encapsulated in the hydrophobic core of the micelle during self‐assembly or they can be directly conjugated to the polymer chain to form a polymer prodrug. They demonstrate high targetability since the hydrophilic ends of the polymers can be highly functionalized with specific ligands.

Encapsulation of EFV in different polymeric micelles formed from both linear triblock polyethylene oxide‐polypropylene oxide‐polyethylene oxide (PEO‐PPO‐PEO) polymers known as poloxamers and four‐arm‐branched PPO chains with PEO blocks at the end of each arm known as poloxamines resulted in improved oral bioavailability of EFV.^89^, ^90^ Poloxamine micelles were found to be less stable than poloxamer micelles, but both micelles led to a more than 5,000 fold increase in EFV solubility from 4 µg/mL to more than 20 mg/mL.^89^ Polymer micelle encapsulation resulted in an 87.6% increase in maximum plasma concentrations of EFV and a 56.5% increase in AUC values in comparison to a suspension of free EFV orally administered in a rat model.^89^ Furthermore, polymer micelles led to a decrease in the variability between individual's pharmacokinetic EFV profiles.^89^ To balance the oral bioavailability with the micelle stability and release kinetics of the different block copolymers, a multi‐component polymer micelle composed of both poloxamers to confer stability and poloxamines to improve EFV encapsulation capacity and oral bioavailability was created.^90^, ^91^ Intranasal administration of these EFV loaded, multi‐component polymer micelles resulted in a CNS concentration of EFV four times higher than intravenous administration of the delivery platform.^91^ Despite their potential for intranasal delivery of targeted drug delivery systems, such polymer micelles are not suitable for use in a sustained release delivery system.^92^ The micelles do not exhibit long‐term release and disassemble upon binding to mucosa, making it necessary to develop an alternative delivery system if sustained release via mucosal administration is a desired function.^92^ It was found that mucosal adhesion could be improved by using a chitosan‐g‐oligo(*N*‐isopropylacrylamide) copolymer to form polymer micelles with the mucoadhesive capabilities of chitosan.^93^ The polymer micelles were stabilized by ionotropically crosslinking the chitosan blocks between different micelles to create a supramolecular nanogel.^93^ This improves the cytocompatibility of the polymer micelles while still enabling the amine groups of chitosan to adhere to the mucosal layer and creates a polymer micelle system potentially capable of sustained drug release.^93^ However, further research is needed into the release profile of the delivery platform and such polymer micelles still exhibit polymer dependent biocompatibility.

#### 3.1.6 Nanosuspensions/drug NPs

Arguably the most successful nanostructured delivery system for ARV delivery in terms of clinical development, with two long‐acting, injectable nanosuspensions of ARV therapeutics in phase III clinical trials,^94^, ^95^ nanosuspensions are gaining attention for the treatment of HIV. They consist of crystallized drugs milled into NPs, stabilized with surfactants, and suspended in an aqueous phase. They can then be injected either subcutaneously or intramuscularly to form a gel‐like drug depot. Because the NPs are pure drug, nanosuspensions have highly efficient drug loading capabilities.^96^ Nanosuspensions are also simple to manufacture in comparison to other nanoscale delivery systems and thus are easy to scale up for mass production.^97^ However, drugs must display the necessary hydrophobicity to be suitable for nanoformulation.^96^, ^97^

Tibotec Pharmaceuticals, in partnership with Johnson & Johnson, developed NPs of varying size of crystalline, free base rilpivirine (RPV) stabilized with different surfactants.^98^ Subcutaneous injection of the 200 nm particles at a dose of 5 mg/kg resulted in constant plasma concentrations of 25 ng/mL for 20 days in dogs with a slow decline to 1–3 ng/mL 3 months after injection.^98^ Further study of the 200 nm RPV nanosuspension revealed that intramuscular injection at a dose of 5 mg/kg led to a 100‐fold higher concentration of RPV in the lymph nodes surrounding the injection site than the plasma one month after injection and a concentration 3–6 fold higher after 3 months.^99^ Phase I clinical trials showed plasma concentrations of 16.2 ng/mL in healthy volunteers 84 days after an intramuscular injection of 600 mg of the long‐acting RPV.^100^, ^101^, ^102^ Following formulation refinement, the nanosuspension currently being explored in phase III clinical trials is a 300 mg/mL formulation of 200 nm RPV free base particles stabilized by the surfactantPoloxamer 338, administered intramuscularly at a dose of 600 mg every 4 weeks or 900 mg every 8 weeks.^103^ ViiV Healthcare, with the support of GlaxoSmithKline, created a nanosuspension of 200 nm NPs of crystalline, free acid cabotegravir stabilized with a surfactant and mixed with an aqueous polymer solution.^96^ Cabotegravir (CAB) is an analog of the FDA approved INSTI dolutegravir currently in phase III clinical trials for long‐acting intramuscular injections.^96^, ^104^ Phase I clinical trials of 100–800 mg doses of the long‐acting CAB suspension injected either subcutaneously or intramuscularly produced plasma concentrations above 0.166 µg/mL (the PA‐IC_90_ value of CAB) for at least 24 weeks for all doses of at least 200 mg and detectable plasma concentrations for all doses at 48 weeks.^96^ Current phase III clinical trials are investigating the efficacy of a 200 mg/mL concentration nanosuspension of 200 nm crystalline, free acid CAB NPs stabilized with polysorbate 20 (the surfactant) and combined in an aqueous solution of PEG 3350 and mannitol, administered intramuscularly at a dose of 400 mg every 4 weeks.^102^

**Figure 5:**
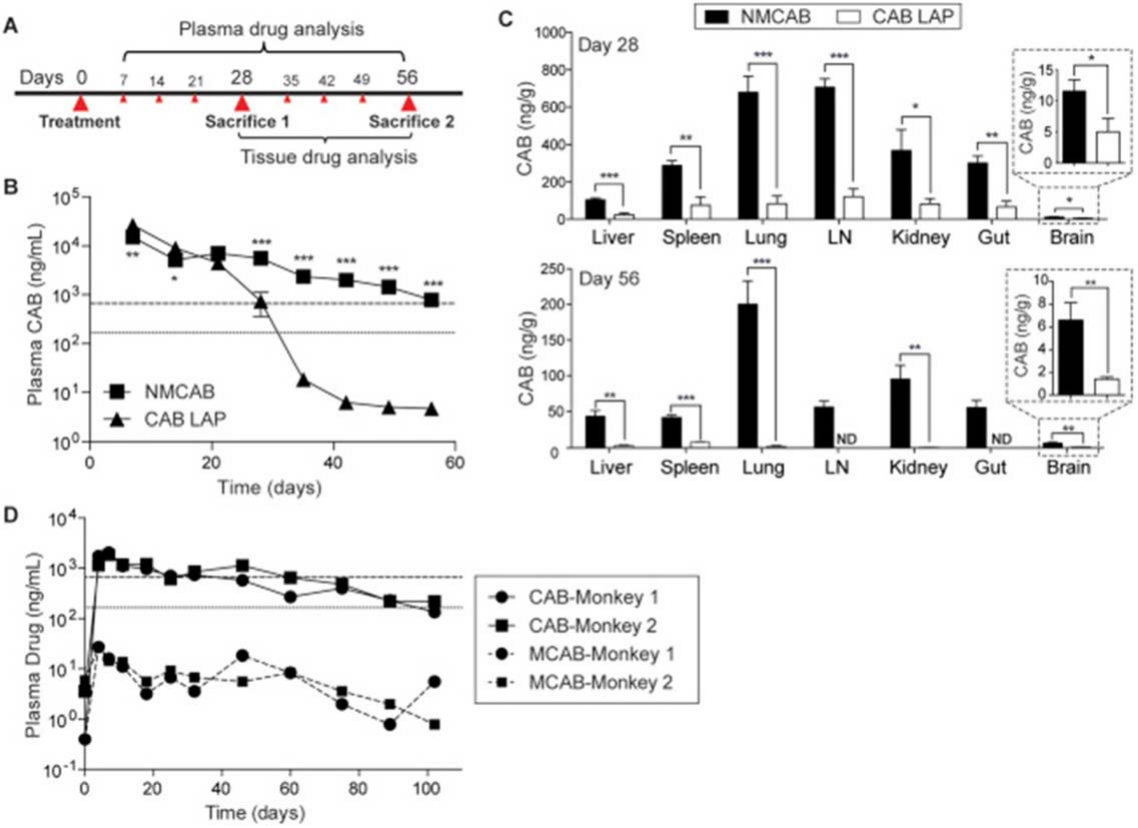
(a) Experimental timeline. (b) Plasma drug concentrations analyzed by UPLC/MS/MS. (c) Tissue drug concentrations analyzed by UPLC/MS/MS. (d) Plasma drug concentrations in the non‐human primate animal models. Used with permission from Ref. 105.

Despite the ability of long‐acting RPV and CAB used in combination to decrease the viral load in mouse animal models and their progression to phase III clinical trials, viral replication was reported in macrophages *in vitro*.^106^ One solution to this lack of penetration is to improve viral reserve accumulation by modifying the nanoformulations to enhance uptake by macrophages.^105^ The Gendelman lab demonstrated improved lymph node and spleen accumulation and increased off‐site accumulation in the liver and lungs of an IDV NP delivered via bone marrow‐derived macrophages in humanized mice.^107^ However, intravenous delivery of foreign macrophages loaded *in vitro* with NPs is not practical for scale up. A more promising approach is the formulation of a myristoylated CAB prodrug that can still crystallize and that can be formed into NPs (NMCAB) via high pressure homogenization.^105^ NMCAB was shown to be more effectively taken up by human monocyte derived macrophage cells *in vitro* than the long‐acting CAB nanosuspension, with NMCAB obtaining an intracellular concentration 60‐fold higher (Figure 5).^105^ Intramuscular injection of NMCAB in mice resulted in CAB concentrations above the PA‐IC_90_ in the lymph nodes, spleen, gut, kidney, and lungs 28 days after the injection whereas all tissue concentrations were below the PA‐IC_90_ value for mice treated with the CAB nanosuspension.^105^ Another challenge is the lack of available long‐acting ARVs for use in combination therapy.^15^ Since RPV and CAB are the only long‐acting formulations currently in clinical development, they are being explored in phase III clinical trials as a combination therapy.^101^ Phase IIb clinical trials of the long‐acting, injectable RPV/CAB therapy demonstrated comparable anti‐HIV activity to an oral regimen consisting of CAB, abacavir, and lamivudine.^104^

It is also possible to create nanosuspensions of RPV and CAB because of their poor water solubility.^15^, ^96^ Most other FDA approved ARVs, including NRTIs, which are a component of almost all FDA approved combination ARV therapeutics, do not have the necessary chemical properties to enable the development of a nanosuspension. If more effective long‐acting combination therapies are to be developed, an alternative approach must be used to create long‐acting delivery systems of NRTIs including TFV, abacavir, emtricitabine, and lamivudine, which are recommended for use in combination with RPV and dolutegravir.

#### 3.1.7 Other nanostructured systems

The nanostructured systems mentioned above are by no means a comprehensive list of all nanoscale systems applied to the treatment of HIV, they are simply the most common systems that have been attempted (with varying levels of success). Carbon nanotubes and quantum dots have also been explored for the treatment of HIV due to their interesting and unique physical and chemical properties. Carbon nanotubes are hollow, cylindrical, single layers of carbon atoms. Spherical fullerene (C_60_) was one of the first NPs shown to have inherent anti‐HIV activity in the early 1990s.^108^ Its therapeutic capabilities were thought to arise from its hydrophobic interactions with the predominantly hydrophobic HIV‐1 protease, which effectively inhibits the activity of the viral enzyme.^108^ However, more recent studies have shown that while fullerene derivatives do inhibit viral maturation, their mechanisms of action are somehow independent of protease inhibition.^109^ Further exploration into carbon nanotubes has shown that the cylindrical version of fullerene also displays anti‐HIV activity due to its high binding affinity for HIV gag proteins and its ability to bind with and alter the confirmation of viral integrase.^110^, ^111^ While the ARV activity of carbon spheres and nanotubes have made them nanomaterials of interest in the treatment of HIV, they have rarely been used in the delivery of ARVs for the treatment of HIV. This lack of use could be due to their insolubility in aqueous environments without further modification that might inhibit their ARV efficacy.^112^ Quantum dots are semi‐conductor nanocrystals that display narrow, tunable emission spectra and that are excitable by a broad range of wavelengths.^113^ Quantum dots rapidly lost their initial popularity as drug delivery vehicles, despite their demonstrated ability to cross the BBB, due to their potential toxicity.^50^ Carbon based quantum dots were recently developed that display enhanced biocompatibility while retaining their BBB permeation capabilities.^50^ There has been increasing use of quantum dots for HIV diagnosis and the monitoring of HIV progression.^114^ One carboxylated quantum dot based system has been functionalized with transferrin and amprenavir (APV) and used to enhance accumulation of the ARV in the brain.^115^ Despite their limited use in the treatment of HIV, quantum dots have the potential to improve diagnosis, progression monitoring, and treatment through their increased accumulation in the brain.

### 3.2 Local delivery systems for HIV prevention

ARVs have also been encapsulated within gel‐forming nanosystems and explored for use as vaginal or rectal microbicides. The goal of such microbicides is local delivery of ARVs to the site of exposure to prevent the acquisition of HIV. The application of nanostructured systems to microbicide formulation can result in prolonged ARV retention at the site of action for sustained transmission protection, increased cellular uptake, protection of microbicide agents from inactivation by the vaginal microenvironment, and improved antiviral activity of encapsulated ARVs.^116^, ^117^, ^118^ Many microbicides are ARV encapsulating, insertable solids or hydrogels made from electrospun polymer fibers, dendrimers, or other polymer NPs. While hydrogels have traditionally been used for local treatment or prevention, there has been recent success with the systemic release of locally delivered hydrogels.^119^ This demonstrates that hydrogels can be used for systemic or local treatment and broadens the scope of use of existing local delivery systems.

#### 3.2.1 Dendrimer based gels

Dendrimers are hyper‐branched, three‐dimensional polymers of well‐defined molecular weight and chain architecture.^120^ They consist of branched polymer arms originating from a multi‐functional core; drugs can be encapsulated within the core, or chemically conjugated onto the terminal ends. Furthermore, it has been shown that cellular uptake mechanism can be controlled based on surface functionalization of the dendrimers, allowing for targeting to specific regions of the cell if desired.^121^ In addition to serving as carriers for therapeutic and imaging agents, dendrimers have also gained attention for use in anti‐HIV microbicides due to the antiviral activity of certain polyanionic dendrimers.^120^, ^122^ Starpharma has developed a naphthalene‐3,6‐disulfonate terminated, polylysine dendrimer named VivaGel with demonstrated HIV preventive abilities in non‐human primate models.^123^, ^124^ Phase I clinical trials for the use of the antiviral microbicide in HIV prevention were completed in 2007 and 2009.^125^, ^126^ The targetability of dendrimers has also been demonstrated by mannosylated, polypropyleneimine, ARV encapsulating dendrimers for systemic delivery that exhibited improved uptake by macrophage and T‐cells and increased anti‐HIV activity.^127^, ^128^ Enhanced BBB penetration and accumulation in microglia (the immune cells of the brain) have been accomplished by loading therapeutics in systemically delivered, hydroxyl‐terminated, polyamidoamine (PAMAM) dendrimers.^129^, ^130^ The mechanism of uptake was altered by changing dendrimer surface charge and size, with 4 nm, neutrally charged, 4‐hydroxyl‐terminated PAMAM dendrimers exhibiting uptake by endocytosis.^130^ The conjugation of other terminal functional groups, such as amines, to PAMAM dendrimers led to hole formation in cellular membranes when dendrimer concentrations were above 10 nM, allowing the dendrimers to diffuse through and enter the cell.^131^ FA was conjugated to the terminal amine groups to create dendrimers capable of targeting cells that express FA receptors.^132^ The described system was initially intended to target epithelial cancer,^132^ but FA receptors are also highly expressed by macrophages and FA targeting has been used in previously described systems to enhance lymph node accumulation of NPs.^87^ While PAMAM dendrimers have not been applied to HIV treatment, their enhanced uptake by microglial cells, their ability to penetrate the BBB when intravenously administered, and the ease with which they can be functionalized with targeting ligands make them attractive candidates for the delivery of ARVs to the CNS. The antiviral nature of dendrimers combined with their targetability to immune cells make them nanostructured systems of interest for both systemic and local delivery.

#### 3.2.2 Polymeric hydrogels

**Figure 6:**
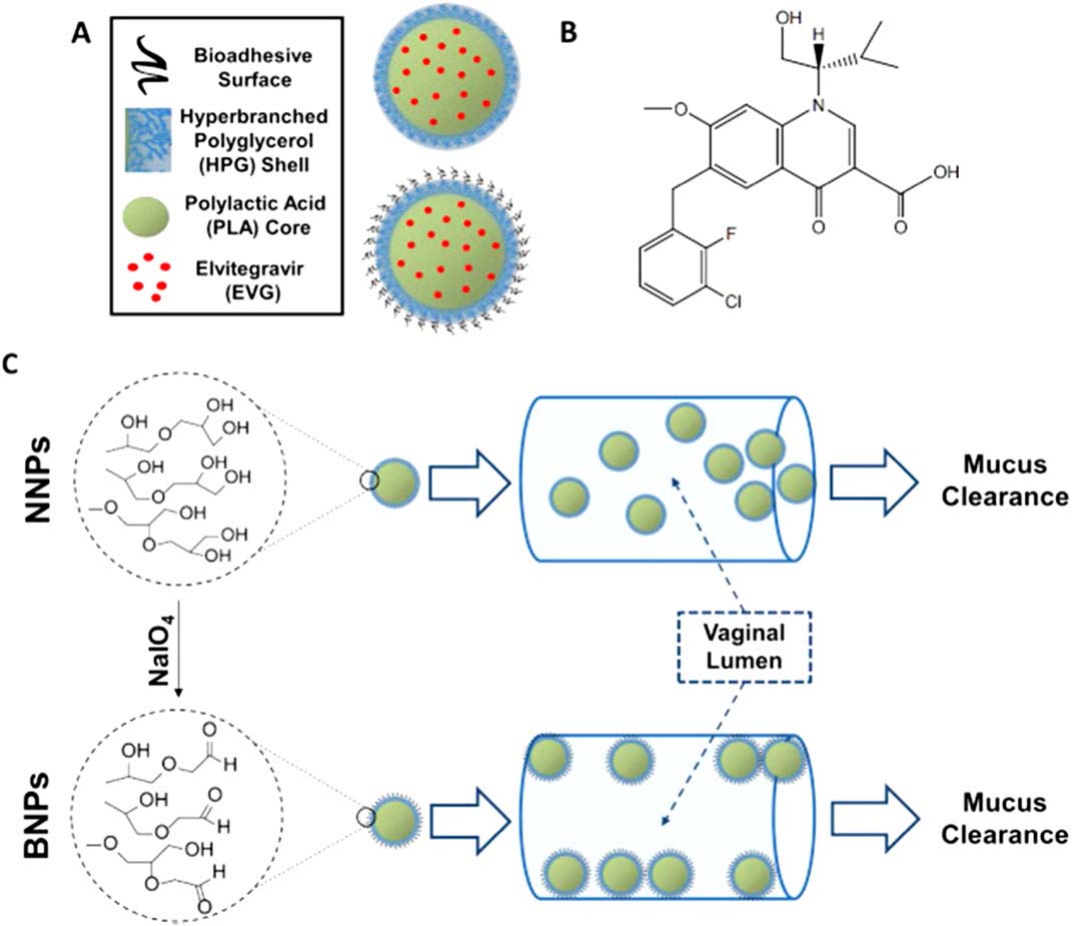
(a) System design of nonadhesive nanoparticles (NNPs) and bioadhesive nanoparticles (BNPs). (b) Chemical structure of elvitegravir. (c) The conversion of NNPs to BNPs using NaIO_4_ and the hypothesized vaginal adherence of the NPs. Used with permission from Ref. 116

Prolonged vaginal retention of ARVs is also important for sustained protection from HIV infection.^116^ The work of the Saltzman lab demonstrated that vaginal retention of elvitegravir can be increased by encapsulating the drug within a PLA matrix coated with surface‐modified, hyper‐branched polyglycerol to form NPs with improved leukocyte and epithelial cell adherence (Figure 6).^116^ A similar strategy is to attach antihuman anti‐CD4 antibodies to the surface of PLGA NPs loaded with SQV and formulated within a hydroxyethylcellulose gel, doubling the uptake of the NPs by CD4+ immune cells following vaginal administration and increasing the ARV concentration at the site of infection.^133^ Incorporation of maraviroc in a silicone elastomer gel produced increased and sustained concentrations of the ARV in the vaginal tissue in comparison to maraviroc loaded hydroxyethylcellulose gel.^134^

#### 3.2.3 Electrospun fibers

Such vaginal gels can have issues of stability and long‐term storage which can be overcome by using solid, electrospun, ARV‐incorporating fibers instead.^118^ Electrospinning of hydrophilic and hydrophobic polymer mixtures containing various ARVs (both hydrophilic and lipophilic in nature) resulted in the formation of drug eluting nanofibers or meshes for use as vaginal microbicides.^135^ It is possible to create electrospun fibers that dissolve rapidly, releasing the loaded ARV in less than 15 min, and fibers that sustain release for more than a week.^118^, ^136^ By combining rapid release hydrophilic core polymers with sustained release hydrophobic shell polymers in coaxial electrospun fibers, precise control of the rate of release of maraviroc from the fibers prevents the burst release commonly observed in uniaxial electrospun fibers.^137^ Altering the core drug loading and the ratio of core to shell thickness of the coaxial spun fibers resulted in drug release that could range from several hours to several days.^137^ The potential for systemic release from local delivery of vaginal microbicides has been demonstrated by administering chitosan to improve local association with specific immune cells and transcytosis of carboxylate‐modified polystyrene NPs through the vaginal epithelium into the female reproductive tract draining lymph nodes, thereby improving both local function of the microbicide and adapting the vaginally administered NPs for systemic function.^138^ While the scalability of ARV loaded electrospun fibers has been demonstrated on production‐scale instruments, few *in vivo* studies have been performed for fiber biocompatibility and there is the potential for residual polymer remnants to remain following use.^118^, ^139^ Furthermore, both dendrimer and electrospun fiber biocompatibility are dependent upon polymer biocompatibility and degradation product toxicity.

## 4 FUTURE PERSPECTIVES

Although nanoscience has shown promise at improving the treatment and prevention of HIV by prolonging the half‐lives of the therapeutics and increasing accumulation in viral reservoir tissues, very few of the proposed nanostructured systems have made it to clinical trials. Many current NP‐based medical approaches face difficulties in part because they do not have the multifunctionality needed to fulfill all the biological and therapeutic requirements.^14^ The capability of nanosystems to improve targeting to viral reservoirs, limit side effects, prolong plasma half‐life, and increase dosing intervals still make them attractive alternatives to traditional oral treatment. However, issues arising from polymer toxicity, poor scalability, limited storage stability, low loading efficiency, and accumulation of nanomaterials in major organs are barriers to the development of the field.^11^ Some have even abandoned nanostructured systems altogether in favor of systems with better potential for progression through clinical trials.

**Figure 7:**
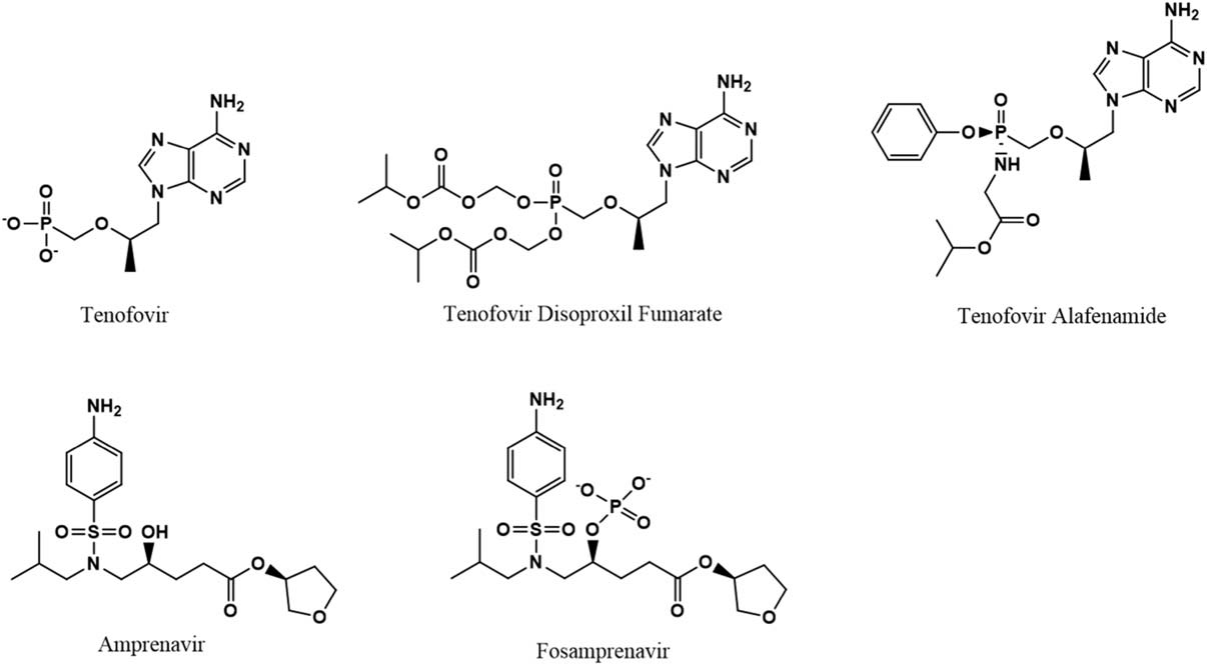
Chemical structures of TFV and APV and their respective prodrugs. The conjugation of acyloxyalkyl esters or aryls to TFV increase its lipophilicity and the addition of l‐alanine isopropyl ester enhances accumulation of the active drug in HIV target cells. The addition of a phosphate group to APV improves its water solubility.

The Langer lab recently reported a millimeter scale, multi‐ARV weekly oral drug delivery device with different arms made from different polymer components with varying degradation rates to control and sustain release for the desired duration.^140^ Another proposed alternative is a sub‐dermal implant of TFV alafenamide capable of releasing 0.92 mg of drug per day with demonstrable zero‐order release kinetics, but that necessitates surgical implantation and removal.^141^ A simpler approach to improve the pharmacokinetic profile of ARVs is to develop prodrugs of the therapeutics with improved bioavailability and cellular uptake. The ARVs TFV and APV are administered solely in their respective prodrug forms of TDF or tenofovir alafenamide (TAF), and fosamprenavir (FPV) (Figure 7).^142^, ^143^ Oral administration of TFV is not possible due to its hydrophilic nature, resulting in poor membrane permeability and subsequently low intestinal absorption.^143^ TDF hides the negatively charged phosphate group and exhibits improved lipophilicity, thereby increasing oral bioavailability.^142^ The aryl and l‐alanine isopropyl ester functionalization of TAF serve a similar purpose as the acyloxyalkyl ester functionalization of TDF, but the phosphonamidate bond of TAF results in preferential accumulation of the drug in HIV target cells.^143^, ^144^, ^145^ APV displays an extremely variable oral bioavailability because of its low water solubility.^142^ FPV has improved water solubility and the prodrug is rapidly converted back to the more membrane permeable APV structure following oral administration.^142^ While TDF, TAF, and FPV are the only FDA approved ARV prodrugs for HIV treatment, prodrugs of emtricitabine have demonstrated promise in preclinical studies.^146^

**Figure 8:**
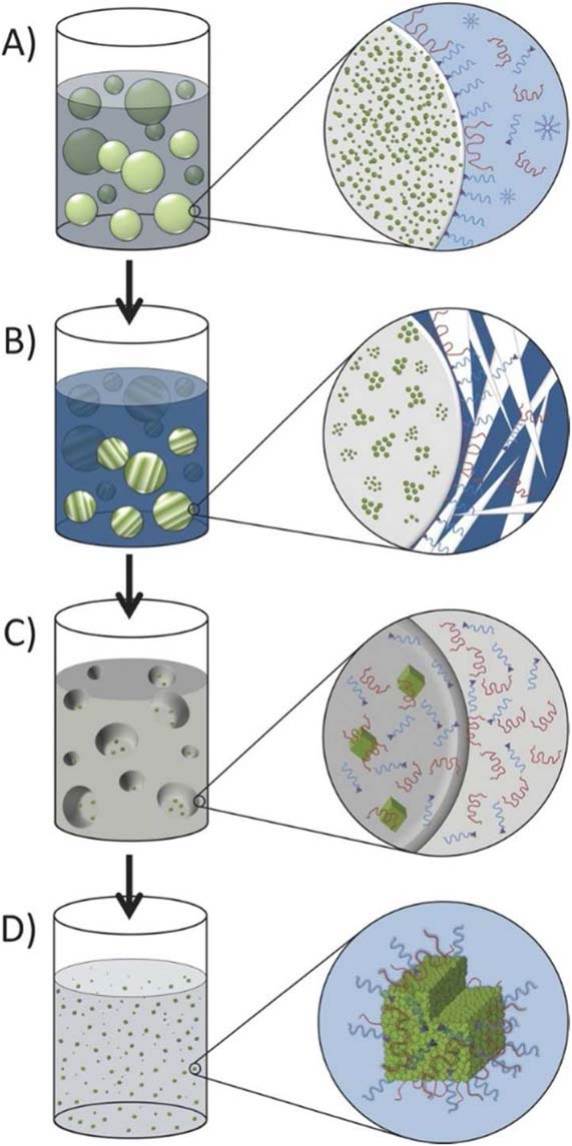
Synthesis of solid drug NPs using emulsion‐templated freeze‐drying. (a) A chloroform‐in‐water emulsion is formed with the hydrophobic drug in the organic phase and the stabilizing surfactants and polymers in the continuous aqueous phase. (b) The growth of ice crystals due to rapid freezing causes supersaturation of the stabilizers and leads to phase separation of the drug and surfactants. (c) Freeze‐drying results in the formation of an emulsion‐templated porous drug monolith surrounded by a matrix of stabilizers. (d) Dissolving the drug monolith and stabilizer matrix in water results in dissolution of the water‐soluble stabilizers and dispersion of the surfactant stabilized, amorphous, solid drug NPs into a nanosuspension. Used with permission from Ref. 150.

There have been some successes in the use of nanostructured systems to formulate ARVs (RPV and CAB), both of which are currently in Phase III clinical trials and expected to enter the market shortly. If they are to be used as long‐acting therapies, other long‐acting ARVs must be developed to use in combination. The Owen lab, in collaboration with the Rannard lab, developed long‐acting nanosuspensions of the NNRTI EFV and the PI LPV that are currently in Phase I clinical trials.^147^ Solid drug NPs of LPV and EFV are formed via emulsion‐templated spray or freeze‐drying and solubilized using surfactants (Figure 8).^148^, ^149^, ^150^ The hydrophobic drugs and stabilizing surfactants are dispersed in a chloroform‐in‐water emulsion, with the drug sequestered in the organic phase and the stabilizers in the continuous aqueous phase.^150^ The emulsion is rapidly frozen, causing the growth of ice crystals and subsequent supersaturation of the surfactants, leading to the phase separation of the drug and the stabilizers.^150^ A porous solid drug particle, surrounded by a matrix of stabilizers, forms after freeze‐drying the frozen emulsion.^150^ Dissolving the drug‐surfactant matrix in water results in the formation of surfactant‐stabilized solid drug NPs approximately 300 nm in size that can be administered orally for daily dosing or intramuscularly for extended release.^148^, ^149^, ^150^ The NPs formed have extremely high drug loading capacities (70 wt% relative to the stabilizers) due to the fact that their primary component is the drug itself.^150^ Oral administration of the solid drug NPs has the potential to reduce the necessary drug dose by up to 50% as a result of increased transport through the gut epithelium and reduced cytotoxicity of the nanoformulation.^148^, ^150^ However, the trial assessing the long‐acting intramuscular administration route was suspended in 2016 due to lack of funding and the formulations face many years of clinical trials before they reach the market.^147^ The development of long‐acting formulations of NRTIs is important, since they are incorporated in all FDA recommended initial regimens,^20^ but no nanostructured systems involving NRTIs have been brought to clinical trials. Since NRTIs do not have suitable chemical properties for development into nanosuspensions, alternative sustained release systems must be explored.

We have extensively explored the use of peptide drug hydrogelators for long‐acting delivery of cancer therapeutics. The formation of amphiphilic peptide prodrugs that self‐assemble into nanofibers and entangle into hydrogels in aqueous environments have been shown to produce controlled release and increased cellular uptake of doxorubicin, paclitaxel, and camptothecin.^151^, ^152^, ^153^ Although ARV hydrogels have been traditionally used for the local delivery of HIV microbicides, the potential for systemic release from locally delivered nanostructured systems has been demonstrated by the injectable ARV nanosuspensions and the codelivery of chitosan with a PS NP microbicide. Based on the successes of TFV, emtricitabine, and APV prodrugs, we believe it is possible to expand these prodrug strategies to other ARVs. Such conversion of hydrophilic NRTIs into amphiphilic peptide prodrugs will promote hydrogel formation *in vivo*. Ideally, hydrogels could be injected intramuscularly or subcutaneously to form a drug depot capable of sustaining systemic release for months. These peptide drug conjugates are simple to synthesize and to scale up, with precisely‐controlled drug loading capacities,^154^ demonstrating the feasibility of creating peptide‐based ARV prodrugs.

## 5 CONCLUSIONS

CART is highly effective at suppressing viral replication in a majority of HIV infected individuals. CART has improved patient survival but the continued presence of the virus in reservoir tissues makes discontinuation of the regimen impossible. Long‐term therapy may be associated with significant adverse side effects and lack of patient compliance to the daily regimen may lead to drug resistance. Consequently, there is a need for an improved delivery system that will prolong dosing intervals, increase accumulation in viral reservoirs, and minimize drug toxicity. Despite numerous examples of increased plasma half‐life and improved viral reservoir penetration of nanostructured systems *in vitro*, preclinical successes have largely not been taken into clinical trials. FDA approved nanoscale delivery systems of ARV therapeutics have not been forthcoming due predominantly to limited scalability and unknown toxicity. To overcome this issue, research efforts must be focused on easily manufacturable drug delivery systems with extensively demonstrated biocompatibility, treatment efficacy, and improved patient compliance.
